# The Gut Microbiota of the Egyptian Mongoose as an Early Warning Indicator of Ecosystem Health in Portugal

**DOI:** 10.3390/ijerph17093104

**Published:** 2020-04-29

**Authors:** Mónica V. Cunha, Teresa Albuquerque, Patrícia Themudo, Carlos Fonseca, Victor Bandeira, Luís M. Rosalino

**Affiliations:** 1 National Institute for Agrarian and Veterinary Research (INIAV, IP), Wildlife, Hunting and Biodiversity R&D Unit, 2780-157 Oeiras, Portugal; teresa.albuquerque@iniav.pt (T.A.); patricia.themudo@iniav.pt (P.T.); 2 Centre for Ecology, Evolution and Environmental Changes (cE3c), Faculdade de Ciências da Universidade de Lisboa, 1749-016 Lisboa, Portugal; lmrosalino@fc.ul.pt; 3 Biosystems & Integrative Sciences Institute (BioISI), Faculdade de Ciências da Universidade de Lisboa, 1749-016 Lisboa, Portugal; 4 Departamento de Biologia & CESAM, Universidade de Aveiro, 3810-193 Aveiro, Portugal; cfonseca@ua.pt (C.F.); victor.bandeira@ua.pt (V.B.)

**Keywords:** *Herpestes ichneumon*, Egyptian mongoose, carnivores, gut microbiota, antimicrobial tolerance, ecosystem health, wildlife management, human health

## Abstract

The Egyptian mongoose is a carnivore mammal species that in the last decades experienced a tremendous expansion in Iberia, particularly in Portugal, mainly due to its remarkable ecological plasticity in response to land-use changes. However, this species may have a disruptive role on native communities in areas where it has recently arrived due to predation and the potential introduction of novel pathogens. We report reference information on the cultivable gut microbial landscape of widely distributed Egyptian mongoose populations (*Herpestes ichneumon*, *n* = 53) and related antimicrobial tolerance across environmental gradients. The panel of isolated species is consistent with the typical protein-based diet of a carnivore: Firmicutes predominate (89% of individuals), while *Clostridiales*, *Enterobacteriales*, and *Lactobacillales* are the major classes. Forty-one individuals (77.4%) harbour *Clostridium* spp. A spatial influence on mongooses’ microbiota is confirmed by nonmetric multidimensional scaling analysis, with a significant contribution of municipality to their microbiota composition. Antimicrobial susceptibility testing of mongoose commensal bacteria to 28 compounds evidences xenobiotic tolerance of *Escherichia coli* (*E. coli*), enterococci, *Salmonella* Spartel and Mbandaka serotypes and *Pseudomonas* bacteria, among others. The common isolation of antimicrobial tolerant microbiota from the mongoose’s gut suggests this species is exposed to anthropogenic influence and is affected by forestry and agricultural-related practices, reflecting its easy adaptation to ecological gradients across agroecosystems. We thus propose regular microbial and phenotypic resistance profiling of widely distributed mongooses as a sentinel tool for xenobiotics’ lifecycle and ecosystem health in Portugal.

## 1. Introduction

The mammalian gut ecosystem is shaped by a complex dynamic interplay between the host’s anatomy, physiology, ecology, and diet [1]. Furthermore, the environment and geographical location have also been shown to deeply influence the composition and abundance of bacterial communities present in the gut [2]. Altogether, these variables contribute to the development of species-specific commensal microbiomes [3].

Commensal bacteria are important in the recruitment and maintenance of energy [4] and the reinforcement of immunity and resistance to pathogens. Conversely, the imbalance of the intestinal microbial community or their abnormal interaction with the immune system may lead to opportunistic infection or other diseases [5,6,7,8]. Hence, the characterization of the microbiota of each species is an opportunity to understand its biology and ecology, and to unravel established associations with microbes, across intraspecific individuals and across natural communities [1,8,9].

Alterations of naturally occurring commensal bacteria may have conservation implications, especially in species already threatened by restricted population size and range. The protective nature of commensal microbiota [10] causes a reduction in microbiome diversity, and richness may decrease overall in immune functions, resulting in higher pathogen incidence and predisposition to illness, which will reduce individuals’ fitness and survivorship—crucial parameters in animal species with reduced population sizes [11]. Moreover, pathogen shifts, spill-over and the development of antimicrobial resistance impose new survival challenges to populations already under extinction pressure, regardless of the taxonomic group. For example, Waite and collaborators [12] detected pathogenic *Pasteurellaceae* in the gut microbiome of the critically endangered kakapo parrot (*Strigops habroptilus*), whose presence they considered an early warning, as *Pasteurella* spp. are frequently found in immunosuppressed and sick individuals. On the other hand, the development of antibiotic resistance in wild microbiota species (e.g., *Escherichia coli* in African mountain gorillas, *Gorilla gorilla beringei* [13]) may endanger any reintroduction program or reduce the success probabilities of clinical treatment of reintroduced or native individuals with illnesses.

In Iberia (Portugal and Spain), as in other regions of the world, the information on faecal microbiota of wild, domestic or captive mammal carnivores is still scarce. The Iberian carnivore guild is varied. It includes the Egyptian mongoose (*Herpestes ichneumon* Linnaeus, 1758), which had a restricted geographic range in the mid 1980s but now is experiencing a rapid expansion, mainly due to its remarkable adaptation to different land uses [14]. In Europe, mongooses are restricted to Iberia. Their rapid and wide range expansion in Portugal in the last 30 years [14,15] due to dispersal capabilities, together with the changes they may possibly induce to the communities, by preying upon vulnerable species [16], could affect native species’ fitness or even survival. This recent expansion into new areas contributed to the selection of this mesocarnivore as a model in this study, in detriment of other common and native species (e.g., red fox, *Vulpes vulpes*), since expanding species may introduce new microbes in formerly pristine areas [17]. Also, several aspects of the mongoose’s biology remain ill-defined, such as population dynamics and the role of biotic (e.g., intra-and inter-specific competition) and abiotic (e.g., climate change) factors.

The mongoose consumes mostly vertebrates, invertebrates and seeds [18,19], is a cursorial predator [18] and, being a trophic and habitat generalist, is expected to reach higher abundances in regions characterized by high human and cattle breeding densities, and high levels of landscape transformation [20]. These greater contact areas and densities, together with their strictly diurnal activity, synanthropic habits [14,21] and certain agricultural practices (e.g., extensive husbandry systems), also facilitate microbial interactions with farm animals, other wildlife species and, ultimately, with humans. Furthermore, the intensive use of antimicrobial agents in food animals may contribute to the emergence and dissemination of resistant bacteria within carnivore populations that may thereafter spill back. Thus, the possible circulation of resistant bacteria within Iberian mesocarnivore populations, and the mongoose in particular, also raises management and conservationist concerns, because this predator has an overlapping distribution with some of Iberia’s most endangered species, including the Iberian lynx (*Lynx pardinus*), the wildcat (*Felis silvestris*), the European polecat (*Mustela putorius*) [22,23] and leporids such as the European rabbit (*Oryctolagus cuniculus*) and the Iberian hare (*Lepus granatensis*) that are facing severe declines due to viral epizootics. Furthermore, antimicrobial resistance emergence in commensal microbiota of widely distributed mammals may serve as an early indicator of the perpetration of xenobiotics in the environment that exert cascading effects on natural communities and thus constitute a risk for ecosystem health.

For these reasons, to improve understanding of the Egyptian mongoose bioecology and the potential risks exerted towards threatened native communities and human health, we defined two interrelated aims in this work. First, to characterize the cultivable commensal bacteria from a Mediterranean population of 53 animals sampled across mainland Portugal; and second, to evaluate mongoose exposure to anthropogenic influence by determining the tolerance of cultivated microbiota to antimicrobials used in animal production and agricultural-related practices, namely at the livestock interface, and to assess microbiota features as indicators of ecosystem health.

## 2. Materials and Methods 

### 2.1. Study Area

**Figure 1:**
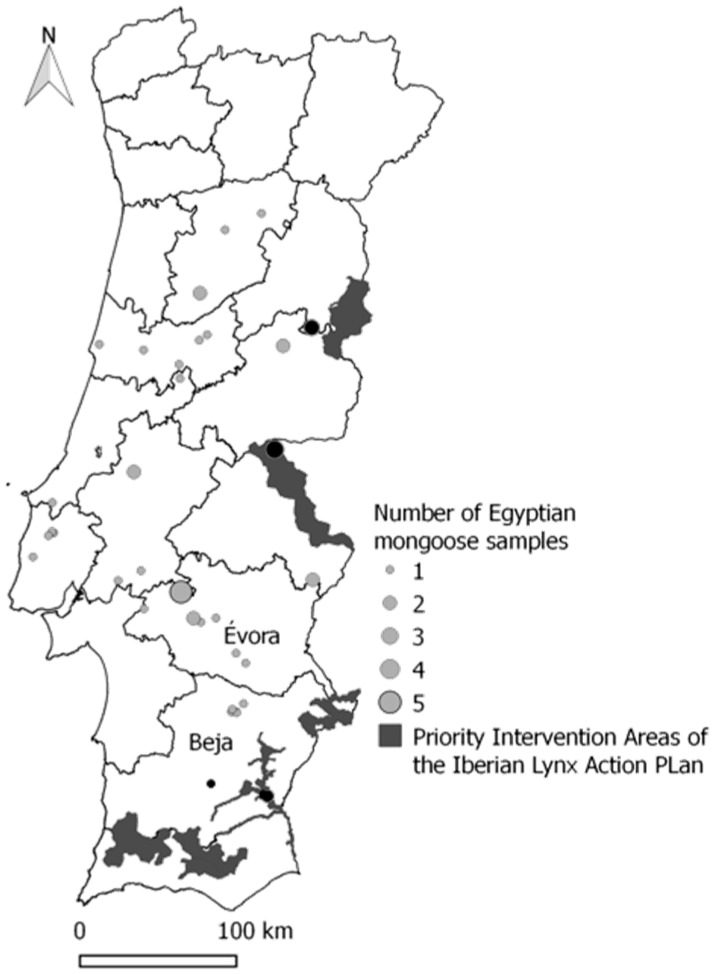
Geographical distribution and sampling range of 53 mongoose specimens. The location of specimens is represented in the map by grey circles. Thirty-five municipalities were sampled. The number of samples with the same GPS coordinates (latitude; longitude) is indicated according to circle diameter, as shown in legend. Black circles represent the specimens located within 15 km of priority intervention areas of the Iberian lynx Action Plan (dark grey areas). Figure produced with open-access software QGIS.

Animals were sampled from 10 out of the 18 districts of mainland Portugal as shown in Figure 1, covering a wide land surface, ranging from low-lying coasts and southern plains to mountain ranges in the north and centre. Despite being relatively small, the Portuguese territory exhibits two main biogeographic areas with considerable climatic contrasts, ranging from Mediterranean summer-dry areas in the south to Atlantic hyper-humid temperate mountains in the northwest [24]. The Mediterranean area is characterized by a mean annual temperature of 18 °C, with mean annual precipitation ranging 275–800 mm, whereas the annual temperature of the Atlantic area is lower than 13 °C and the precipitation ranges 1400–3200 mm (Instituto de Metereologia, http://www.meteo.pt).

### 2.2. Animal and Faeces Samples Collection

Fifty-three Egyptian mongoose (*Herpestes ichneumon*) carcasses, donated for scientific purposes by road technicians or hunters, were collected from 35 municipalities between 2008 to 2011, from accidental road-kills (*n* = 3) or legal game management actions aimed at controlling predator densities (*n* = 50). Ethical approval for this study is not applicable as it did not involve the sacrifice of any animals for the specific purposes herein described. Samples were opportunistically collected from specimens gathered by third parties in the context of legal hunting or accidental run-over. After being geographically located and collected on roads, or after hunting sessions, animal carcasses were frozen at −20 °C until necropsy. The carcasses were thawed and necropsied, and the abdominal cavity of each specimen was opened and the intestines isolated. Solid intestinal content was collected from the rectum of each animal, using a sterile faeces collection tube, and immediately processed for further analysis.

All animals showing clear signs of putrefaction were excluded from this analysis. No gross lesions in the gastrointestinal tract or signs of clinical disease were detected at necropsy, even though histopathological examinations performed in the scope of another study evidenced partial autolysis in a subset of road killed specimens. Faeces of all specimens were moulded, without visible signs of diarrhoea.

### 2.3. Bacterial Isolation, Biochemical and Molecular Identification, Serotyping and Virulence Genes

Each faecal sample was thawed immediately before proceeding to cultivation, to preserve both aerobic and anaerobic species and to avoid potential loss of bacterial viability and composition changes. Bacterial cultivation and isolation were performed using standard media and enrichment techniques in accordance to established routine procedures by the National Reference Laboratory (NRL) for animal diseases, Portugal (INIAV I.P.). Briefly, approximately 5 g of each faecal sample was removed aseptically from the container, suspended in sterile saline and homogenized. This suspension was inoculated onto four different solid media with an inoculation loop. For anaerobic bacteria isolation, inoculation was performed on Schaedler agar supplemented with 5% sheep blood (bioMérieux, Marcy-l’Étoile, France) and incubated for 48 h under the absence of oxygen and strict anaerobiosis, using atmosphere generation systems (Anaerogen, Thermo Fisher Scientific Oxoid Ltd., Basingstoke, UK).

The faeces suspension was also inoculated onto Trypticase Soy Agar supplemented with 5% sheep blood (TSS) (bioMérieux, Marcy-l’Étoile, France) and onto MacConkey plates (bioMérieux, Marcy-l’Étoile, France). All the plates were incubated at 37 °C, for 24 h, under the presence of oxygen. If bacterial growth was not observed within 24 h, incubation was extended up to 72 h.

One to three colonies with distinctive morphology or size, grown from each faecal sample on each medium, were subcultured onto fresh media and characterized using Gram stains, selective media, biochemical tests, and identification kits. The colonies grown on TSA were subcultured into Veal Infusion Broth (Becton Dickinson, Thermofisher, Basingstoke, UK), supplemented with glucose and horse serum (Invitrogen), and followed by a smear and Gram stain. All the microorganisms grown on MacConkey were subcultured into Triple Sugar Iron (TSI) and tested for oxidase screening. Based on macro and microscopic morphology and Gram staining characteristics exhibited by the isolates obtained on the different media, phenotypic identification proceeded through biochemical characterization using the API^®^ test strips (ID 32 STREP, API CORYN, API 50 CHB, ID 32E, ID 32GN, ID 32A) (bioMérieux, Marcy-l’Étoile, France), according to established algorithms routinely used in INIAV IP. Quality control strains were used to interpret and validate each test batch, according to the specifications indicated by the manufacturer. Results reading and interpretation were done with the aid of the ATB™ Expression reading system (version 2.0, bioMérieux, Marcy-l’Étoile, France). Phenotypic identifications were accepted for precision superior to 99.5%. In some cases, additional tailor-made biochemical tests were performed on an isolate level (oxidase, urease, catalase, indole, and proline arylamidase, among others). If the identification by API of a subset of isolates from the same host showing identical macro and microscopic morphology yielded the same taxon, then one random isolate was selected among those for further testing and statistical analysis.

For *Salmonella* spp. detection, the standard ISO6579 (attach D) was followed. A pre-enrichment of the faeces suspension (1 mL) was initially performed in 9 mL of buffered peptone water (Merck, Kenilworth, NJ, USA) at 37 °C for 18 ± 2 h, prior to selective enrichment in Rappaport–Vassiliadis (MSRV) medium (Merck, Darmstadt, Germany) at 41.5 °C for 24/48 h. Subsequently, the selective media *XLD* agar (bioMérieux, Marcy-l’Étoile, France) and chromID™ *Salmonella* (bioMérieux, Marcy-l’Étoile, France) were inoculated and incubated at 37 °C for 24 h. The identification of characteristic colonies was confirmed through biochemical identification using ID 32E (bioMérieux, Marcy-l’Étoile, France). Serotyping was performed according to the Kauffmann–White scheme and Grimont and Weill (2007) [25].

*Escherichia coli* isolates obtained on MacConkey plates and subcultured into TSI were identified according to classical biochemical procedures and using ID20E commercial strips. The identity of one isolate from each bacterial genus or species assigned by means of *API* galleries was also determined at the molecular level using PCR targeting the eubacterial 16S rDNA gene, with the primers and conditions described by Marchesi et al. (1998) [26]. Eighteen representative isolates of phenotypic variants were selected for partial 16S rDNA sequencing. DNA extraction was performed using a commercial system (High Pure PCR Template Preparation Kit, Roche, Basel, Switzerland) following the manufacturer’s instructions. After amplification of the 16S rDNA region and electrophoresis on a 1.0% agarose gel containing ethidium bromide, the PCR products were excised, purified using a commercial kit (QIAquick gel extraction kit; Qiagen, Hilden, Germany) and commercially sequenced (GATC Biotech, Germany). Original chromatogram files were inspected and manually reviewed. Edited sequences were compared using BLAST software (megablast) with similar reference sequences available in GenBank (http://www.ncbi.nlm.nih.gov). Sequences were annotated with taxonomic information from the top three best matches displaying the same nucleotide pairwise identity. Isolates with ≥99% identity were annotated at the species level, 97% to <99% identity were annotated at the genus level; 95% to <97% identity were annotated at the family level; and isolates with <95% identity were annotated at the order level. Members of the family *Enterobacteriaceae* with identities of 95–99% were annotated at the family level and members of the family *Pseudomonadaceae* spp. with identities of 95–99% were classified at the genus level (Supplementary Material).

The detection of *E. coli attaching and effacing* gene (*eae*) and of genes encoding heat-labile enterotoxin (Lt), heat-stable enterotoxin (Sta), verocitotoxin (Vtx1, Vxt2), and the subtypes of verocitotoxin (Vtx1a, Vtx1c, Vtx1d, Vtx2a, Vtx2c, Vtx2d), were performed by PCR according to the methodologies described by the EU Reference Laboratory for *E. coli* and based on [27,28,29,30,31,32,33,34,35,36,37]. The strains used as controls in the PCR assays were *E. coli* ED647 (positive control for *vt1*, *vt2*, *eae* genes), *E. coli* EDL 933 (*vtx1a*), *E. coli* DG 131/3 (*vtx1c*), *E. coli* MHI 813 (*vtx1d*), *E. coli* 94C (*vtx2a*), *E. coli* O31 (*vtx2c*), *E. coli* C 165-02 (*vtx2d*), *E. coli* NN14 (*Sta*; *Lt*), and *E. coli* JM-109 (negative control).

### 2.4. Antimicrobial Susceptibility Testing 

The antimicrobial susceptibilities of all the isolates were determined using the automated ATB™ (bioMérieux, Marcy-l’Étoile, France) susceptibility testing system. This commercial method is based on microdilution and includes test strips adapted for bacteria of veterinary origin (ATB VET^®^, bioMérieux, Marcy-l’Étoile, France), with an array of 28 antimicrobials indicated in Table 1. Growth of pre-inoculums, inoculation and incubation on antimicrobial galleries were performed following the manufacturer’s guidelines and under conditions similar to the agar dilution or microdilution methods. The density of bacterial suspensions was adjusted to 0.5 McFarland, depending on the growth of bacterial species, and incubated for 18–24 h at 37 °C, under aerobiosis or anaerobiosis, or in a CO_2_-enriched atmosphere, according to the isolate characteristics. Gallery reading, based on the presence of bacterial growth as indicated by turbidity, and classification of the strain as sensitive or resistant were done automatically with the ATB™ Expression reading system (bioMérieux, Marcy-l’Étoile, France). Strains *Escherichia coli* ATCC 25922, *Staphylococcus aureus* ATCC 25923, *Enterococcus faecalis* ATCC 29212, *Pseudomonas aeruginosa* ATCC 27853 and *Streptococcus pneumoniae* ATCC 49619 were included as quality controls.

### 2.5. Statistical Analysis of Data

We determined bacterial flora diversity index (α) as an indication of genera abundance, which was calculated as α = [(S − 1) × 0.4343]/log N], wherein S is the total number of genera and N is the total number of bacterial isolates [38]. We also tested microbiota richness spatial autocorrelation to assess possible data spatial clustering, using Moran’s I index [39]. We then used nonmetric multidimensional scaling (NMS) analysis to perform an ordination of the microbiota community present in sampled mesocarnivores, using Bray–Curtis dissimilarity measures. NMS is an ordination technique with few assumptions regarding data characteristics (e.g., no assumption of data linearity, no restriction on data distance measures) and therefore is adequate for a wide type of data [40]. We used it to assess which factors might contribute the most to the similarities between an individual’s microbiota communities. We tested the influence of sample location (municipality) and individual sex and age on individual microbiota community composition. For all statistical analysis, we used R software [41], together with its packages “Vegan” [42] and “ape” [43].

We tested the differences in antimicrobial tolerance by comparing the overall and between bacterial species mean resistance of isolates, using Kruskal–Wallis (K) and Mann–Whitney (U) tests, after Lilliefors (Kolmogorov-Smirnov) normality tests confirmed data skewness [44].

## 3. Results

### 3.1. Dominant Cultivable Microbiota

Eighty-two phenotypically different bacterial isolates were recovered from the faeces of surveyed specimens in selective media, which contained a relatively homogeneous microbiota dominated by Gram-positive bacteria (64%). Based on biochemical identification, we identified twenty-three bacterial species belonging to fourteen genera, classified into three phyla: *Firmicutes* (57% of bacterial species), *Proteobacteria* (30%) and *Actinobacteria* (13%). One to four bacterial species were isolated from each animal specimen. Eighty-nine percent of mongoose samples contained at least one *Firmicutes* bacterial species. At the individual level, bacteria affiliated within the order *Clostridiales* predominated (*n* = 41, 77.4%), followed by *Lactobacillales* (24.5%), *Enterobacteriales* (20.8%) and *Pseudomonadales* (13.2%). *Actinomycetales* and anaerobic *Bacillales* were only sporadically isolated (7.6%, each), while *Caulobacterales* and *Sphingomonodales* were rare (1.9%, each). Alpha-proteobacteria were a minor constituent of the microbial community (Table 1). *Enterococcus* was the second most represented genus (four different species), after *Clostridium* (five species), as shown in Table 1. *Clostridium sordellii* was the most frequently isolated microorganism (*n* = 24 animals; 45.3%), followed by *C. clostridioforme*, *C. perfringens*, *E. coli* and *Pseudomonas putida* that were each present in the faeces of more than 10% of surveyed mongooses, as displayed in Table 1. Three *Salmonella* strains were isolated from three mongoose specimens, exhibiting serotypes Spartel and Mbandaka and serotype [II 1, 4, [5], 12, [27]:b:[e,n,x] (1)]. Among *E. coli* isolates, PCR analyses revealed that none of the strains carried the *eae* gene or any of the genes coding for heat-labile enterotoxin, heat-stable enterotoxin, verocitotoxin and verocitotoxin subtypes.

Ten of the 53 mongooses were collected next (within 15 km) to priority intervention areas of the Iberian lynx Action Plan, as shown in Figure 1. These specimens harboured *Clostridium* spp. [(100%, including *C. sordellii* (80%)], *Enterococcus* spp. (three specimens), *Salmonella* spp. (two specimens) and *E. coli*.

No age or sex effects on the cultivable microbiota from different mongoose specimens were apparent. We did not detect a significant spatial autocorrelation in phenetic microbiota diversity (Moran’s I = 0.03, *p* = 0.490). However, NMS results show a significant contribution of municipality where the samples were collected to the similarity between individual microbiota communities (Supplementary Tables S1 and S2 and Supplementary Figure S1). The NMS model’s stress value indicates a good two-dimensional configuration of the data (Stress = 0.099) [40].

Sequencing of 16S rDNA amplicon from 18 representative bacterial phenetic species, according to biochemical and API tests, generated partial sequences of various lengths located in the initial 500-bp of the gene comprising the V3-V4 variable region, a polymorphic moiety that usually provides adequate differentiation for identification of most genera. Alignment of annotated with similar publicly available reference sequences generated pairwise nucleotide identities ranging from 89% to 99%, as shown in Supplementary Table S3. To compare agreement between phenotypic and 16S rDNA classification, we based phylotype assignment on information from the top three best matches using the criteria specified in the methods section. Pairwise nucleotide identity analyses delivered results at the order and family levels consistent with traditional phenotypic classification for all but three isolates (agreement of 83%), as shown in Supplementary Table S3. In most cases, the layer of taxonomic classification could be established at genus level.

### 3.2. Antimicrobial Resistance Phenotypes 

**Figure 2:**
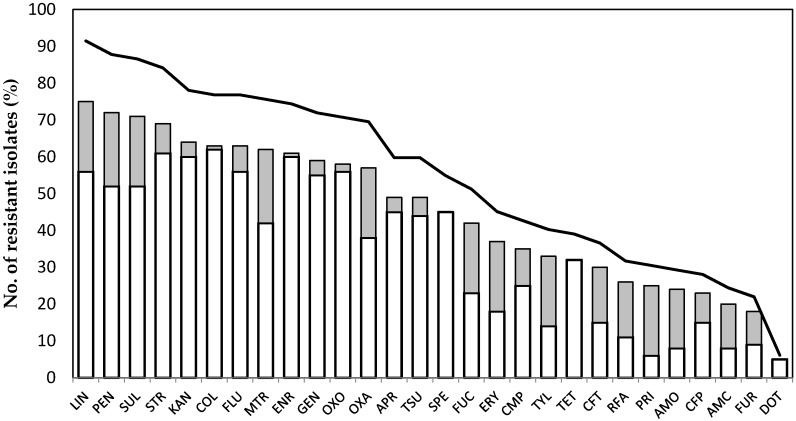
Resistance of bacterial isolates to 28 antimicrobials. The bars represent the number of Gram-negative (in grey) and Gram-positive (in white) isolates exhibiting resistance to each antimicrobial, while the line represents the percentage of all resistant isolates. Legend: Amoxicillin–clavulanic acid (AMC), Amoxicillin (AMO), Apramycin (APR), Cefoperazone (CFP), Cephalothin (CFT), Colistin (COL), Chloramphenicol (CMP), Doxycycline (DOT), Erythromycin (ERY), Enrofloxacin (ENR), Flumequin (FLU), Fusidic acid (FUC), Nitrofurantoin (FUR), Gentamicin (GEN), Kanamycin (KAN), Lincomycin (LIN), Metronidazol (MTR), Oxacillin (OXA), Oxolinic Acid (OXO), Penicillin (PEN), Pristinamycin (PRI), Rifampicin (RFA), Spectinomycin (SPE), Streptomycin (STR), Sulfamethizole (SUL), Tetracycline (TET), Cotrimoxazole (TSU), Tylosin (TYL).

The susceptibilities of the bacterial species cultivated from mongooses presented a remarkable variation to the 28 antimicrobial agents tested (K = 73.458, df = 22, *p* < 0.001; Table 1, Figure 2). A snapshot summary of the antibiotic compounds to which each binomial “phenetic/phylogenetic” bacterium was resistant or susceptible is also provided in supplementary material (Supplementary Table S4).

**Figure 3:**
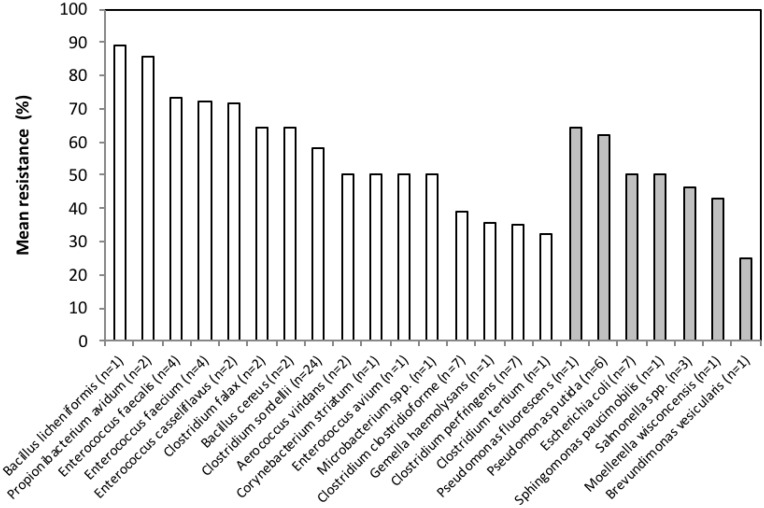
Mean percentage of antimicrobial agents to which the isolates within each bacterial species are resistant. The white bars represent Gram-positive species while Gram-negative are presented in grey.

Considering phenetic classifications, *Bacillus licheniformis* and *Propionibacterium avidum* isolates were among the most widely resistant (over 85% of the antimicrobials tested), followed by enterococci, which were able to grow in the presence of compounds from at least five antimicrobial classes, as shown in Table 1, Figure 2 and Figure 3 and Supplementary Table S4). Among the Gram-negative, *Pseudomonas* isolates apparently were the least susceptible, with no significant differences in antimicrobial tolerance between species (U = 457, *p* = 0.529). At the tested concentrations, more than 57% of the antimicrobials were unable to inhibit the growth of more than 50% of the isolates, as shown in Figure 2.

In addition, more than 84% of the isolates were resistant to lincomycin, penicillin, streptomycin or sulfamethizole. In contrast, less than 7% of the isolates grew in the presence of doxycycline, as shown in Figure 1. Colistin was ineffective towards all Gram-positive bacteria, to which they are naturally resistant [45], and with the exception of *Moellerella wisconcensis*, all Gram-negative isolates were susceptible. Likewise, spectinomycin, tetracycline, and doxycycline, inhibited the growth of all Gram-negative bacteria, as practically did enrofloxacin and oxolinic acid; only the *P. fluorescens* strain was resistant to quinolones and fluroquinolones. At the tested concentration, enrofloxacin did not impair the growth of the majority of Gram-positive bacteria; four *C. perfringens* and one *B. cereus* isolates did not grow in the presence of flumequin or oxolinic acid, as shown in Table 1 and Figure 2.

All isolates affiliated within *Bacillus*, *Pseudomonas* and *Sphingomonas* genera were resistant to the six β-lactams tested. With the exception of cephalotin and cephoperazone cephalosporines, the other beta-lactams were unable to inhibit the growth of *Salmonella*, *A. viridans*, *C. striatum*, and *Microbacterium* spp. isolates, as well as one *Enterococcus faecalis* isolate. Penicillin, to which Enterobacteriaceae are inherently resistant [45], was the least effective β-lactam, with only 12.2% of the isolates (mostly *Clostridium perfringens* and *Propionibacterium avidum*) being inhibited, as shown in Table 1 and Figure 2.

In contrast to Gram-negative and enterococcal isolates, Gram-positive bacilli were generally susceptible to macrolide drugs and pristinamycin. Apart from the natural resistance of enterococci [45] and a few other Gram-positive species, Gram-negative isolates were, in general, susceptible to the aminoglycosides tested; the exceptions were four *E. coli* and *Salmonella* isolates that grew in the presence of streptomycin, as displayed in Table 1.

Strikingly, metronidazole-and fusidic acid-resistant *Clostridium sordelli* isolates were highly prevalent (79.2% and 75%, respectively); *C. falax* also being resistant, while all *C. perfringens* isolates were susceptible, as shown in Table 1. No significant difference in the mean resistance of *Clostridium* (K = 8.579, df = 4, *p* = 0.073) and *Enterococcus* isolates (K = 4.606, df = 3, *p* = 0.203) to the 28 tested antimicrobials were detected.

## 4. Discussion

In this study, the panel of cultivable commensal bacteria of a large population of the Egyptian mongoose was assessed, providing reference data for a widespread species whose biology remains to be uncovered in many aspects. Moreover, the panel of characterized mongoose samples originated from several areas in mainland Portugal, representing a variety of ecological gradients with different primary productivity indices and, thus, contrasting availability and diversity of food items which could influence microbiota composition, as well as different patterns of human pressure. Our aim to generate baseline data for mongoose microbiota on a population level in detriment of highlighting individual microbiota led us to mainly follow a nonselective bacterial isolation approach that was appropriate for the cultivation of nonfastidious and fastidious aerobes and anaerobes. For that purpose, Schaedler agar supplemented with sheep blood was especially useful for the recovery of anaerobic bacteria, as was TSA blood agar that is a general purpose medium for nonfastidious bacteria, also enabling growth of more demanding and fastidious groups; in addition, seeding on MacConkey enabled cultivation and differentiation of lactose fermenting from nonfermenting Gram-negative. Our study revealed low phylum-level diversity consisting almost exclusively of *Firmicutes* and *Proteobacteria*. We also did not find as much bacterial diversity as theoretically would be expected for an opportunistic carnivore. At the genus level, bacterial α diversity index was 2.95. In other carnivores from other regions of the world, for instance grizzly and black bears that have omnivorous diets, α diversity indices from rectal swabs ranged from 2.69 to 4.10 [46]. Culture-dependent methods could limit the detection of some uncultivable or stressed bacterial groups, underestimating bacterial diversity [47]. In addition, taking into consideration the opportunistic origin of our samples, the period ranging from death to faecal sample collection might have reduced the abundance and diversity of cultivable microbiota at the individual sample level. Culture-based surveys are available for sympatric carnivore species from different biotopes, for instance the otter (*Lutra lutra*), in which an α diversity index of 6.55 can be extrapolated from reported data [48]. However, we cannot infer how the bacterial diversity within mongooses compares to sympatric carnivores with similar lifestyle, as no culture-based studies have been reported for other species. Nevertheless, by picking phenotypically different colonies in both selective and unselective media, a representative collection of bacteria was achieved, and the main bacterial phyla recovered from the mongooses were relatively concordant among individuals. We thus speculate that the panel of isolated bacteria most probably represents the cultivable core microbial community of the intestines of mongooses.

The microbiota from the specimens surveyed was dominated by Gram-positive bacteria, mainly of the phylum *Firmicutes* (detected in 89% individuals), with *Clostridium* species, particularly *C. sordelli*, being the most common microorganism among mongoose specimens (77% and 45%, respectively). High-protein contents have been reported to select for proteolytic bacteria and, particularly, for *Clostridium* populations [49,50], which is corroborated by the carnivorous diet of mongooses [16,18,19]. Enterococci and *Escherichia coli*, which are common inhabitants of the intestinal tract of mammals, were also isolated. *E. faecium* and *E. faecalis* were the most prevalent enterococcal species (73%) among enterococci-positive faecal samples (21%), which is in agreement with other wildlife studies focused on the otter, badger or fox [51,52,53]. Millán and coworkers (2009) [54], working in a Spanish area where Iberian lynxes occur (Doñana), detected active infections with *Salmonella enterica* in 12% of mongooses. To confirm if *Salmonella* could also be present in mongooses sampled from Portugal, in addition to our main nonselective cultivation strategy, we used a protocol with selective media that is specific for *Salmonella* isolation. Although we isolated several *Salmonella* serotypes, evidences of generalized clinical disease or enteric infection were not found, leading us to hypothesize that mongooses may be asymptomatic carriers of *Salmonella* spp. and that their presence might possibly be related with the diet of mongooses. Mbandaka serotype is often isolated from broilers and feedstock in Portugal. The serotype II 1, 4, [5], 12, [27]:b:[e,n,x] has been isolated from pork meat delicatessen, the environment, and cold blooded animals (unpublished data). It is thus natural to speculate that the isolation of these *Salmonella* strains probably reflects the diet of mongooses and their opportunistic nature. Our results show that microbiota richness (i.e., number of phenotypic species detected in each sample) is not affected by samples’ geographical location. However, NMS results indicated that microbiota similarity between individual mesocarnivores was significantly affected by the municipality where the samples were collected. This pattern indicates that animals living in closer proximity to each other have similar microbiota communities, but not in term of species richness, as shown by the absence of significant spatial autocorrelation. So, our findings rather support a spatial influence on microbiota communities’ composition, leading us to hypothesize that Mediterranean habitat characteristics, primary productivity, and thus available food resources in a given location, may influence an individual’s microbiota. In agreement with these findings, a study by Chen and collaborators (2020) [55] evidenced that the feeding environment had important effects on the faecal microbiota of spotted hyenas (*Crocuta Crocuta*). The role of anthropogenic influence on agroecosystems with an indirect impact on mongoose microbiota is also likely, making microbiota assessment an informative tool to evaluate an ecosystems’ health.

We identified several bacterial groups that may be zoonotic or have a pathogenic role for other animals, such as those immunocompromised by coinfections [56]. In addition, several bacterial groups are known to survive for long periods in the environment, favouring transmission by the faecal–oral route or via contaminated water, food or the environment. For instance, the presence and potential excretion of *Clostridium* pathogenic strains, whose virulence is attributed to numerous exotoxins, may carry the risk for lethal enteritis and enterotoxaemia infections in cattle and sheep. In other carnivores, *Clostridium sordellii* has been responsible for the sudden death of captive lions (*Panthera leo*) [56], while *Clostridium perfringens* has been associated to perforating enterocolitis in captive cheetahs (*Acinonyx jubatus*) [57]. The presence of *E. coli* in American river otters (*Lutra canadensis*) has been related with genitourinary infections [58] and there are records of salmonellosis in Eurasian badgers (*Meles meles*) from England [59]. It is also well established that despite being commensals, both enterococci and *E. coli* may potentially carry transferable resistance genes and virulence determinants [60]. Nevertheless, among the *E. coli* strains isolated in this study, toxin-encoding genes and the *eae* gene were not detected.

The tolerance to antimicrobials of different classes detected among the commensal bacteria isolated from mongooses is remarkable: the isolates from seventeen (74%) of overall cultivable bacterial species were, on average, tolerant to the majority of antimicrobials. Among enterococci, extensive antimicrobial tolerance was also evident, but no significant differences were observed among the four species isolated. In Portugal, the detection of antimicrobial resistant isolates has been documented for several wild carnivores, such as the threatened Iberian wolf (*Canis lupus*) [61,62] and the otter [48]. Until now, there is no clear indication on how these carnivores have acquired such resistant strains, with some authors considering that the general pattern would be for wildlife to harbour naturally low resistant bacteria [51,63]. Other authors argue that there is cumulating evidence for some relevant resistance genes to have been originated in environmental microbes [64]. Thaller and collaborators [65] support the view that acquired antibiotic resistance is more highly associated with anthropic pressure and, consequently, exposure to antibiotics, rather than with ecological and landscape conditions. Thus, although mongooses may contribute to the dispersal of such resistant strains, especially because they are considered to be expanding their range [14], we hypothesize that they may also be affected by agricultural-related practices and be a powerful indicator of ecosystem health. In areas were agriculture and cattle production are the main activities, as those of rural Iberia, antimicrobials are extensively used to optimize animal health and production. As mongooses are also scavengers [16], and their distribution overlaps livestock farms, by consuming domestic animals or small mammals or by sharing food and water placed in devices aimed at feeding domestic or game species (such as wild rabbit or red-legged partridge)—a common practice in Iberia, they become exposed to livestock production antimicrobials, or anthelminthic, leading to the emergence of resistant strains. Furthermore, mongooses may also intersect ponds and small reservoirs used by cattle to feed and drink, especially in the dry Mediterranean climate areas where water shortage is acute in summer. Cattle often defecate and urinate in these places, which may be a source for exposure to antimicrobial compounds and for nonwild type bacterial strains [66]. In agreement with these assumptions, we isolated multiresistant Gram-negative *P. putida* and *Salmonella* spp., both recognized as opportunist pathogens of humans and animals, from mongoose specimens sampled in 2010 in Beja and Évora regions. We hypothesize that the resistance traits of the microbiota isolated from mongooses may, at least in part, reflect selection under pressure of coexistence with antimicrobials related to agriculture practices.

The circulation and spread of resistant bacteria throughout the ecosystem may represent a health problem for sympatric endangered species and spill-back to livestock and humans. Egyptian mongooses have bioecological characteristics that may facilitate intra-and inter-specific pathogen transmission, such as defecation in latrines, some of them communal and used as scent marking stations by different individuals [67]. Moreover, this predator is known by its cursorial habits, patrolling territories that might reach 3.10 km^2^ [68]. This ranging behaviour, together with its abundance (1.2 individuals/km^2^ in Spain; [69]), may facilitate intra-and inter-specific pathogen spread and horizontal transfer of resistance and virulence determinants. The synergic effect of both of these factors may have an impact on areas of direct and indirect interactions with humans and also on areas of sympatry with threatened species.

The detection of antimicrobial resistant strains in the vicinity of habitat areas of endangered species, together with recently published data indicating the extensive circulation of feline panleukopenia virus and *Mycobacterium avium* subsp. *paratuberculosis* within mongoose populations [70,71], support the recommendation to continue to evaluate the sanitary condition of wildlife in areas where Iberian lynx and other protected species have been individually released. Moreover, pathogen exposure effects might be exacerbated when acting synergistically with other factors, such as malnutrition, stress, or inbreeding [32]. The combined effect of pathogen shifts and antimicrobial resistance occurrence on species already compromised by reduced population sizes, small genetic diversity pools and reduced overall immunity and fitness, make them highly susceptible to stochastic events such as epidemic outbreaks [11,17].

## 5. Conclusions

The presence of inherently resistant bacteria or with acquired resistance mechanisms in the intestinal tract of expanding mongoose populations is relevant in mammalian ecology, wildlife management and conservation, and human health. Overall, although mongooses may contribute to the dispersal of resistant strains, especially because they are considered to be expanding their range at the livestock–wildlife interface, we hypothesize that they may also be affected by agricultural-related practices that impact animals’ health and welfare, regardless of the mammal species. Hence, mongoose microbiota might be a powerful indicator for ecosystem health in the Iberia area, particularly in Portugal where this study was developed, and an early warning indicator for agents circulating at the human–livestock–wildlife interface.

## Figures and Tables

**Table 1:** Mean resistance of isolates within each bacterial species to 28 antimicrobials. The number of isolates tested per species is indicated in brackets. Amoxicillin–clavulanic acid (AMC), Amoxicillin (AMO), Apramycin (APR), Cefoperazone (CFP), Cephalothin (CFT), Colistin (COL), Chloramphenicol (CMP), Doxycycline (DOT), Erythromycin (ERY), Enrofloxacin (ENR), Flumequin (FLU), Fusidic acid (FUC), Nitrofurantoin (FUR), Gentamicin (GEN), Kanamycin (KAN), Lincomycin (LIN), Metronidazol (MTR), Oxacillin (OXA), Oxolinic Acid (OXO), Penicillin (PEN), Pristinamycin (PRI), Rifampicin (RFA), Spectinomycin (SPE), Streptomycin (STR), Sulfamethizole (SUL), Tetracycline (TET), Cotrimoxazole (TSU), Tylosin (TYL).

**Phylum**	**Bacterial Phenotypic Species (no. Isolates)**	**PEN**	**AMO**	**AMC**	**OXA**	**CFT**	**CFP**	**STR**	**SPE**	**KAN**	**GEN**	**APR**	**CMP**	**TET**	**DOT**
*Firmicutes*	*Aerococcus viridans* (*n* = 2)	100	100	100	100	0	0	100	0	100	0	0	0	100	0
*Firmicutes*	*Bacillus cereus* (*n* = 2)	100	100	100	100	100	100	100	50	50	50	50	0	100	50
*Firmicutes*	*Bacillus licheniformis* (*n* = 1)	100	100	100	100	100	100	100	100	100	100	100	0	100	100
*Firmicutes*	*Clostridium clostridioforme* (*n* = 7)	100	0	0	0	0	0	100	0	100	100	0	0	0	0
*Firmicutes*	*Clostridium falax* (*n* = 1)	100	0	0	0	0	0	100	100	100	100	100	100	0	0
*Firmicutes*	*Clostridium perfringens* (*n* = 7)	43	0	0	0	29	0	100	71	100	100	100	0	29	0
*Firmicutes*	*Clostridium sordellii* (*n* = 24)	88	0	0	75	0	0	100	96	100	100	92	75	79	0
*Firmicutes*	*Clostridium tertium* (*n* = 1)	100	0	0	0	0	0	100	100	100	100	100	0	0	0
*Actinobacteria*	*Corynebacterium striatum* (*n* = 1)	100	100	100	100	0	0	100	0	100	0	0	0	100	0
*Firmicutes*	*Enterococcus avium* (*n* = 1)	100	0	0	100	100	100	100	100	100	0	0	0	0	0
*Firmicutes*	*Enterococcus casseliflavus* (*n* = 2)	100	0	0	100	100	100	100	100	100	100	100	0	0	0
*Firmicutes*	*Enterococcus faecalis* (*n* = 4)	100	25	25	100	75	75	100	75	100	75	75	75	25	0
*Firmicutes*	*Enterococcus faecium* (*n* = 4)	100	0	0	100	100	100	100	100	100	100	100	0	0	0
*Firmicutes*	*Gemella haemolysans* (*n* = 1)	0	0	0	0	0	0	0	0	0	100	0	0	100	100
*Actinobacteria*	*Microbacterium* spp. (*n* = 1)	100	100	100	100	0	0	100	0	100	0	0	0	100	0
*Actinobacteria*	*Propionibacterium avidum* (*n* = 2)	0	0	0	100	0	100	100	100	100	100	100	100	100	100
*Proteobacteria*	*Brevundimonas vesicularis* (*n* = 1)	100	100	100	0	0	0	0	0	0	0	0	0	0	0
*Proteobacteria*	*Escherichia coli* (*n* = 7)	100	43	0	100	100	0	57	0	57	57	57	57	0	0
*Proteobacteria*	*Moellerella wisconcensis* (*n* = 1)	100	100	0	100	0	0	0	0	0	0	0	0	0	0
*Proteobacteria*	*Pseudomonas fluorescens* (*n* = 1)	100	100	100	100	100	100	100	0	0	0	0	100	0	0
*Proteobacteria*	*Pseudomonas putida* (*n* = 6)	100	100	100	100	100	100	0	0	0	0	0	83	0	0
*Proteobacteria*	*Salmonella* spp. (*n* = 3)	100	100	100	100	0	0	100	0	0	0	0	0	0	0
*Proteobacteria*	*Sphingomonas paucimobilis* (*n* = 1)	100	100	100	100	100	100	0	0	0	0	0	0	0	0
**Phylum**	**Bacterial Phenotypic Species (no. Isolates)**	**ERY**	**LIN**	**PRI**	**TYL**	**COL**	**TSU**	**SUL**	**FLU**	**OXO**	**ENR**	**FUR**	**FUC**	**RFA**	**MTR**
*Firmicutes*	*Aerococcus viridans* (*n* = 2)	0	100	0	0	100	0	100	100	100	100	0	0	0	100
*Firmicutes*	*Bacillus cereus* (*n* = 2)	0	100	0	0	100	100	100	50	50	50	0	100	0	100
*Firmicutes*	*Bacillus licheniformis* (*n* = 1)	0	100	100	100	100	0	100	100	100	100	100	100	100	100
*Firmicutes*	*Clostridium clostridioforme* (*n* = 7)	0	100	0	0	100	100	100	100	100	100	0	0	0	0
*Firmicutes*	*Clostridium falax* (*n* = 1)	100	100	0	100	100	100	100	100	100	100	0	0	100	100
*Firmicutes*	*Clostridium perfringens* (*n* = 7)	57	43	0	0	100	14	14	43	43	100	0	0	0	0
*Firmicutes*	*Clostridium sordellii* (*n* = 24)	0	96	0	0	100	83	88	100	100	100	0	75	0	79
*Firmicutes*	*Clostridium tertium* (*n* = 1)	100	100	0	0	100	0	0	0	0	0	0	0	0	0
*Actinobacteria*	*Corynebacterium striatum* (*n* = 1)	0	100	0	0	100	0	100	100	100	100	0	0	0	100
*Firmicutes*	*Enterococcus avium* (*n* = 1)	0	0	0	0	100	100	100	100	100	100	0	0	0	100
*Firmicutes*	*Enterococcus casseliflavus* (*n* = 2)	100	100	0	100	100	100	100	100	100	100	100	0	0	100
*Firmicutes*	*Enterococcus faecalis* (*n* = 4)	75	100	75	75	100	75	100	100	100	100	75	0	50	100
*Firmicutes*	*Enterococcus faecium* (*n* = 4)	100	100	0	100	100	100	100	100	100	100	25	0	100	100
*Firmicutes*	*Gemella haemolysans* (*n* = 1)	0	100	0	0	100	0	100	100	100	100	0	0	0	100
*Actinobacteria*	*Microbacterium* spp. (*n* = 1)	0	100	0	0	100	0	100	100	100	100	0	0	0	100
*Actinobacteria*	*Propionibacterium avidum* (*n* = 2)	100	100	100	100	100	100	100	100	100	100	100	100	100	100
*Proteobacteria*	*Brevundimonas vesicularis* (*n* = 1)	0	0	0	0	0	0	100	100	100	0	0	0	0	100
*Proteobacteria*	*Escherichia coli* (*n* = 7)	100	100	100	100	0	0	100	0	0	0	0	100	100	100
*Proteobacteria*	*Moellerella wisconcensis* (*n* = 1)	100	100	100	100	100	0	100	0	0	0	100	100	0	100
*Proteobacteria*	*Pseudomonas fluorescens* (*n* = 1)	100	100	100	100	0	0	0	100	100	100	100	100	0	100
*Proteobacteria*	*Pseudomonas putida* (*n* = 6)	100	100	100	100	0	83	100	83	0	0	100	100	83	100
*Proteobacteria*	*Salmonella* spp. (*n* = 3)	100	100	100	100	0	0	100	0	0	0	0	100	100	100
*Proteobacteria*	*Sphingomonas paucimobilis* (*n* = 1)	100	100	100	100	0	0	100	0	0	0	100	100	0	100

## Supplementary Materials

The following are available online at https://www.mdpi.com/1660-4601/17/9/3104/s1, Table S1: Centroids of each factor tested for the two dimensions of the Nonmetric Multidimensional Scaling model (NMDS1 and NMDS2), resulting from fitting the environmental vectors. Table S2: Goodness of fit of the nonmetric multidimensional scaling (NMS) analysis. Table S3: Information on the partial 16S rDNA nucleotide sequences of a selected group of isolates. Table S4: Antimicrobial phenotypes of the selected isolates whose 16S rDNA was partially sequenced. Figure S1: Spatial representation of the location of sampled animals (open circles) and microbiota bacterial species using the two dimensions of the Nonmetric Multidimensional Scaling model (NMDS1 and NMDS2).
