# Supplementary material for: The Gut Microbiota of the Egyptian Mongoose as an Early Warning Indicator of Ecosystem Health in Portugal
Source: Int J Environ Res Public Health. 2020 Apr 29;17(9):3104. doi: 10.3390/ijerph17093104 (PMC7246908; doi:10.3390/ijerph17093104)
Supplement: Supplementary file 1 [file ijerph-17-03104-s001.pdf]

SUPLEMMENTARY MATERIAL

Table S1. Centroids of each factor tested for the two dimensions of the Nonmetric Multidimensional Scaling model (NMDS1 and NMDS2), resulting from fitting the environmental vectors.

Factors	NMDS1	NMDS2
as.factor(Sex) Female	−0.0794	0.0248
as.factor(Sex) Male	0.0525	0.0657
As.factor (Age) Adult	−0.0084	0.0568
As.factor (Age) Juvenile	0	−0.0158
as.factor(Municipality) Évora	0.4119	−0.1094
as.factor(Municipality) Alenquer	0.5232	0.1469
as.factor(Municipality) Anadia	0.4187	−0.0196
as.factor(Municipality) Arganil	0.1009	0.2381
as.factor(Municipality) Arruda dos Vinhos	0.5300	0.1836
as.factor(Municipality) Beja	−0.1131	−0.2363
as.factor(Municipality) Benavente	−0.2779	0.1706
as.factor(Municipality) Bombarral	0.1009	0.2381
as.factor(Municipality) Castanheira de Pêra	0.1497	−0.6986
as.factor(Municipality) Castro d'Aire	0.0628	−0.1935
as.factor(Municipality) Coimbra	−0.4390	−0.4639
as.factor(Municipality) Coruche	0.4837	0.1811
as.factor(Municipality) Elvas	−0.2307	0.2944
as.factor(Municipality) Figueira da Foz	0.9749	0.1797
as.factor(Municipality) Fundão	0.3685	−0.6260
as.factor(Municipality) Guarda	−0.5480	0.3575
as.factor(Municipality) Idanha-a-Nova	0.0022	0.2850
as.factor(Municipality) Lousã	0.7573	−0.0951
as.factor(Municipality) Mértola	0.4187	−0.0196
as.factor(Municipality) Mafra	0.1009	0.2381
as.factor(Municipality) Marinha Grande	0.5082	0.1341
as.factor(Municipality) Montemor-o-Novo	−0.1086	0.0978
as.factor(Municipality) Nisa	0.6779	0.0640
as.factor(Municipality) Oeiras	0.1009	0.2381
as.factor(Municipality) Portel	−0.4241	0.2416
as.factor(Municipality) Sabugal	−0.4013	−0.1078
as.factor(Municipality) Sertã	0.1556	−0.3531
as.factor(Municipality) Tábua	−0.1168	−0.1502
as.factor(Municipality) Tondela	−0.5758	0.2231
as.factor(Municipality) Torres Novas	−0.2432	−0.0289
as.factor(Municipality) Torres Vedras	−0.0081	0.3872
as.factor(Municipality) Vendas Novas	0.5232	0.1469
as.factor(Municipality) Vidigueira	0.4119	−0.1094
as.factor(Municipality) Vila Velha de Ródão	−0.7307	−0.1082
as.factor(Municipality) Viseu	−0.2688	0.0860

Table S2. Goodness of fit of the nonmetric multidimensional scaling (NMS) analysis.

	R^2	*p*-Value
**Sex**	0.015	0.314
**Age**	0.019	0.856
**Municipality**	0.582	0.039

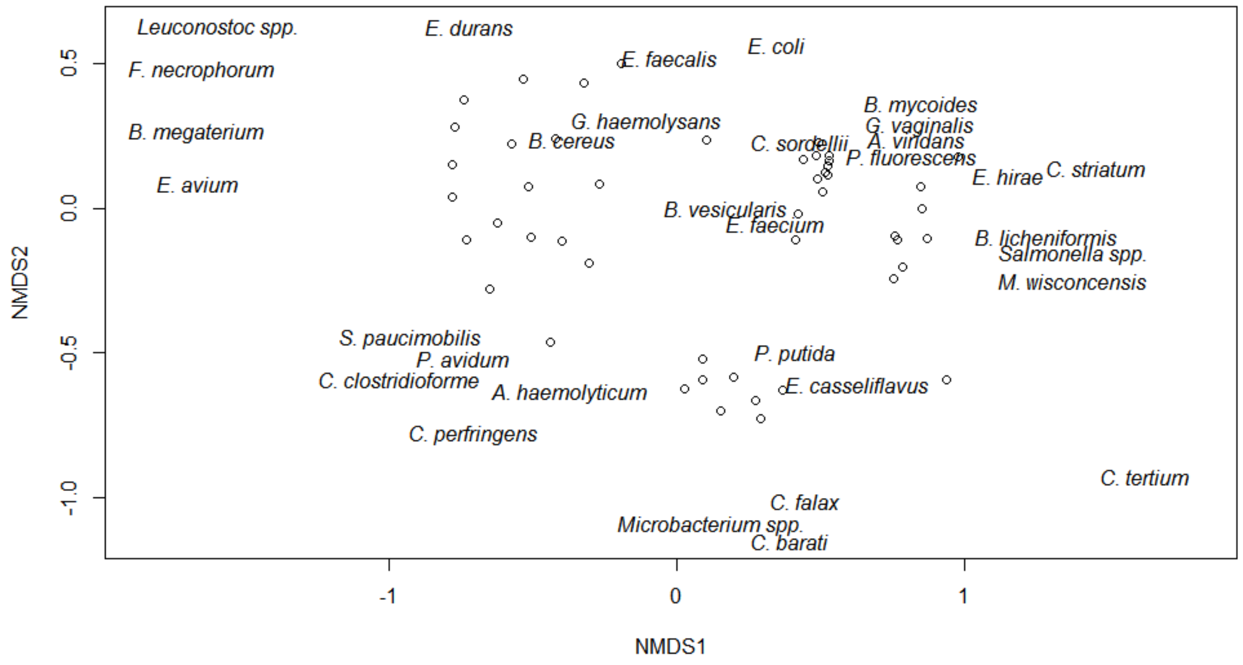

Figure S1. Spatial representation of the location of sampled animals (open circles) and microbiota bacterial species using the two dimensions of the Nonmetric Multidimensional Scaling model (NMDS1 and NMDS2). Also see Table 1 for phenotypic-based taxonomic classification.

Table S3. Information on the partial 16S rDNA nucleotide sequences of a selected group of isolates.

Mongoose ID	Isolate ID	Phenotypic Bacterial Identification#	Nucleotide Sequence Length	Closest Reference Sequence Match (1st hit)	Accession Number	Query cover	Evalue	Nucleotide Sequence Identity	Assigned Phylotype** (Order, family, genus, or species)
8215 (1)	4	*Salmonella*† spp.	282 bp	*Salmonella enterica* subsp. *enterica* serovar *Typhimurium* strain NCIM 2501 16S ribosomal RNA gene, partial sequence	KR078393.1	100%	7e-129	97%	*Enterobacteriaceae*
				Salmonella enterica subsp. *enterica* serovar *Enteritidis* strain SEE2, complete genome	CP011791.1	100%	7e-129	97%	
				Salmonella enterica subsp. *enterica* serovar *Senftenberg*	LN868945.1	100%	7e-129	97%	
15858 (1)	7	*Enterococcus casseliflavus*	131 bp	*Enterococcus* sp. S22 16S ribosomal RNA gene, partial sequence	FJ892743.1	98%	4e-48	94%	Lactobacillales
				Enterococcus durans partial 16S rRNA gene, isolate JC318	LN829662.1	98%	2e-47	94%	
				Enterococcus faecium strain Vm3 16S ribosomal RNA gene, partial sequence	HM638426.1	98%	2e-47	94%	
22124 (5)	9	*Clostridium clostridioforme*	129 bp	*Clostridium vulturis* strain YMB-57 16S ribosomal RNA gene, partial sequence	JQ423949.2	96%	2e-46	95%	*Clostridiaceae*
22124 (9)	11	*Pseudomonas putida*	360 bp	*Pseudomonas* sp. 145P5BR 16S ribosomal RNA gene, partial sequence	KR611622.1	99%	0.0	99%	*Pseudomonas* sp.
				Pseudomonas deceptionensis strain DC5 16S ribosomal RNA gene, partial sequence	KR338996.1	99%	0.0	99%	
				Pseudomonas fragi strain 11–06 16S ribosomal RNA gene, partial sequence	KJ817447.1	99%	0.0	99%	
28005 (8)	15	*Moellerella*† *wisconcensis*	384 bp	*Moellerella wisconsensis* strain E3–4 16S ribosomal RNA gene, partial sequence	KP058388.1	99%	4e-101	97%	*Moellerella* sp.
28005 (9)	18	*Escherichia*† *coli*	408 bp	*Escherichia* sp. UIWRF0784 16S ribosomal RNA gene, partial sequence	KR189900.1	100%	0.0	99%	*Enterobacteriaceae*
				Shigella sonnei strain U3H 16S ribosomal RNA gene, partial sequence	KM658277.1	100%	0.0	99%	
				Escherichia fergusonii strain CCFM8346 16S ribosomal RNA gene, partial sequence	KJ803903.1	100%	0.0	99%	
41220 (2)	19	*Clostridium perfringens*	1001 bp	*Clostridium perfringens* strain JP55, complete genome	CP010993.1	99%	0.0	99%	*Clostridium perfringens*

				Clostridium perfringens ATCC 13124, complete genome	CP000246.1	99%	0.0	99%	
				Clostridium perfringens DNA, complete genome, strain: CBA7123	AP017630.1	99%	0.0	99%	
887 (3)	29	*Enterococcus avium*	170 bp	*Enterococcus durans* strain IMAU60173 16S ribosomal RNA gene, partial sequence	FJ917727.1	81%	8e-42	90%	Lactobacillales
				Enterococcus sp. S22 16S ribosomal RNA gene, partial sequence	FJ892743.1	81%	3e-41	90%	
				Enterococcus faecium strain Vm3 16S ribosomal RNA gene, partial sequence	HM638426.1	81%	1e-40	90%	
887 (1)	30	*Pseudomonas fluorescens*	252 bp	*Pseudomonas* sp. CC6K 16S ribosomal RNA gene, partial sequence	KM187301.1	100%	2e-124	99%	*Pseudomonas* sp.
				Pseudomonas grimontii strain IHB B 13622 16S ribosomal RNA gene, partial sequence	KP762550.1	100%	2e-124	99%	
				Pseudomonas brenneri strain HG1–1B 16S ribosomal RNA gene, partial sequence	KM891558.1	100%	2e-124	99%	
13580 (3)	34	*Bacillus licheniformis*	247 bp	*Bacillus cereus* gene for 16S ribosomal RNA, partial sequence	AB709908.1	97%	1e-121	99%	*Bacillus* sp.
13580 (3)	35	*Enterococcus faecium*	985 bp	*Enterococcus sp.* 692B1_12AGalle 16S ribosomal RNA gene, partial sequence	KU644404.1	99%	0.0	99%	*Enterococcus hirae*
				Enterococcus hirae strain RCB815 16S ribosomal RNA gene, partial sequence	KT261027.1	100%	0.0	99%	
				Enterococcus hirae strain BR8-B(B) 16S ribosomal RNA gene, partial sequence	MF498499.1	99%	0.0	99%	
17705 (4)	114	*Bacillus cereus*	272 bp	*Bacillus cereus* strain NCIM 2155 16S ribosomal RNA gene, partial sequence	KR078401.1	99%	2e-135	99%	*Bacillus* sp.
				Bacillus thuringiensis serovar indiana strain HD521, complete genome	CP010106.1	99%	2e-135	99%	
				Bacillus sp. Hin7.3 16S ribosomal RNA gene, partial sequence	KP962330.1	99%	2e-135	99%	
886 (4)	26	*Enterococcus faecalis*	992 bp	*Enterococcus faecalis* strain L1C21R1 16S ribosomal RNA gene, partial sequence	KX373594.1	100%	0.0	99%	*Enterococcus faecalis*
				Enterococcus faecalis strain L1C21M8 16S ribosomal RNA gene, partial sequence	KX373593.1	100%	0.0	99%	
				Enterococcus faecalis strain L1C21M6 16S ribosomal RNA gene, partial sequence	KX373591.1	100%	0.0	99%	

22124 (5)	10	*Microbacterium spp*	123 bp	*Carnobacterium sp.* strain CP_44 16S ribosomal RNA gene, partial sequence	KY041845.1	87	5e-43	97%	*Carnobacterium* sp‡
				Carnobacterium sp. enrichment culture partial 16S rRNA gene, clone SB3	KR055032.1	87%	5e-43	97%	
				Carnobacterium sp. enrichment culture partial 16S rRNA gene, clone SB7	LT009680.1	87%	5e-43	97%	
28005 (8)	16	*Brevundimonas vesicularis*	236 bp	*Carnobacterium* sp. SLS1-P10 16S ribosomal RNA gene, partial sequence	KR023932.1	100%	3e-127	99%	*Carnobacterium* sp‡
28005 (2)	17	*Aerococcus viridans*	220 bp	Uncultured *Carnobacterium* sp. clone S4_D12 16S ribosomal RNA gene, partial sequence	KP181813.1	54%	1e-30	89%	Lactobacillales
886 (4)	24	*Corynebacterium striatum*	117 bp	*Weissella* sp. B 235 16S ribosomal RNA gene, partial sequence	GU998859.1	100%	4e-48	97%	*Weissella* sp‡
				Weissella viridescens gene for 16S ribosomal RNA, partial sequence, strain: JCM 1174	LC065037.1	100%	5e-47	97%	
				Weissella confusa strain 3214O2 16S ribosomal RNA gene, partial sequence	KF598909.1	100%	5e-47	97%	
14981 (9)	100	*Gemella haemolysans*	372 bp	Uncultured bacterium clone Herring_AG02 16S ribosomal RNA gene, partial sequence	JQ191427.1	93%	7e-170	98%	N/A

# Based on API, other biochemical tests, and serotyping (performed for *Salmonella* sp. and *Escherichia coli* only);

* This study; N/A apply to sequences with length less than 200 nucleotides that are not accepted by Genbank.

** Isolates with ≥99% identity with closest reference sequence matches were annotated at the species level whenever the top three hit sequences with the same nucleotide pairwise identity were concordant at all levels of taxonomic classification; 97% to <99% identity were annotated at the genus level; 95% to <97% identity were annotated at the family level; and isolates with <95% identity were annotated at the order level. Members of the family Enterobacteriaceae† with identities of 95%–99% were annotated at the family level. *Pseudomonas* isolates with identities of 95%–99% were annotated at the genus level. Isolates for which there was disagreement between phenotypic and 16S rRNA-based identification are marked with ‡. N/A not attributed;

Table S4. Antimicrobial phenotypes of the selected isolates whose 16S rDNA was partially sequenced.

Animal ID	Isolate ID	Phenotypic ID	Gram	Antimicrobial Phenotype																											
				PEN	AMX	AMC	OXA	CFT	CFP	STR	SPE	KAN	GEN	APR	CMP	TET	DOT	ERI	LIN	PRI	TIL	COL	COT	SUL	FLU	OXO	ENR	NIT	FUS	RIF	MET
28005 (2)	17	*Aerococcus viridans*	+	R	R	R	R	S	S	R	S	R	S	S	S	R	S	S	R	S	S	R	S	R	R	R	R	S	S	S	R
17705 (4)	114	*Bacillus cereus*	+	R	R	R	R	R	R	R	R	R	R	R	S	R	S	S	R	S	S	R	R	R	R	R	R	S	R	S	R
13580 (3)	34	*Bacillus licheniformis*	+	R	R	R	R	R	R	R	R	R	R	R	S	R	R	S	R	R	R	R	S	R	R	R	R	R	R	R	R
22124 (5)	9	*Clostridium clostridioforme*	+	R	S	S	S	S	S	R	S	R	R	S	S	S	S	S	R	S	S	R	R	R	R	R	R	S	S	S	S
41220 (2)	19	*Clostridium perfringens*	+	S	S	S	S	S	S	R	R	R	R	R	S	S	S	R	S	S	S	R	S	S	S	S	R	S	S	S	S
886 (4)	24	*Corynebacterium striatum*	+	R	R	R	R	S	S	R	S	R	S	S	S	R	S	S	R	S	S	R	S	R	R	R	R	S	S	S	R
887 (3)	29	*Enterococcus avium*	+	R	S	S	R	R	R	R	R	R	S	S	S	S	S	S	S	S	S	R	R	R	R	R	R	S	S	S	R
15858 (1)	7	*Enterococcus casseliflavus*	+	R	S	S	R	R	R	R	R	R	R	R	S	S	S	R	R	S	R	R	R	R	R	R	R	R	S	S	R
886 (4)	26	*Enterococcus faecalis*	+	R	R	R	R	S	S	R	S	R	S	S	S	R	S	S	R	S	S	R	S	R	R	R	R	S	S	S	R
13580 (3)	35	*Enterococcus faecium*	+	R	S	S	R	R	R	R	R	R	R	R	S	S	S	R	R	S	R	R	R	R	R	R	R	S	S	R	R
14981 (9)	100	*Gemella haemolysans*	+	S	S	S	S	S	S	S	S	S	R	S	S	R	R	S	R	S	S	R	S	R	R	R	R	S	S	S	R
22124 (5)	10	*Microbacterium spp*	+	R	R	R	R	S	S	R	S	R	S	S	S	R	S	S	R	S	S	R	S	R	R	R	R	S	S	S	R
18465 (2)	119	*Propionibacterium avidum*	+	S	S	S	R	S	R	R	R	R	R	R	R	R	R	R	R	R	R	R	R	R	R	R	R	R	R	R	R
28005 (8)	16	*Brevundimonas vesicularis*	-	R	R	R	S	S	S	S	S	S	S	S	S	S	S	S	S	S	S	S	S	R	R	R	S	S	S	S	R
28005 (9)	18	*Escherichia coli*	-	R	R	S	R	R	S	S	S	S	S	S	S	S	S	R	R	R	R	S	S	R	S	S	S	S	R	R	R
28005 (8)	15	*Moellerella wisconcensis*	-	R	R	S	R	S	S	S	S	S	S	S	S	S	S	R	R	R	R	R	S	R	S	S	S	R	R	S	R
887 (1)	30	*Pseudomonas fluorescens*	-	R	R	R	R	R	R	R	S	S	S	S	R	S	S	R	R	R	R	S	S	S	R	R	R	R	R	S	R
22124 (9)	11	*Pseudomonas putida*	-	R	R	R	R	R	R	S	S	S	S	S	S	S	S	R	R	R	R	S	S	R	S	S	S	R	R	S	R
8215 (1)	4	*Salmonella spp*	-	R	R	R	R	S	S	R	S	S	S	S	S	S	S	R	R	R	R	S	S	R	S	S	S	S	R	R	R
